# Analysis of the characteristics of cracked teeth and evaluation of pulp status according to periodontal probing depth

**DOI:** 10.1186/s12903-017-0434-x

**Published:** 2017-11-28

**Authors:** Sung-Eun Yang, A-Ra Jo, Hye-Jin Lee, Sin-Young Kim

**Affiliations:** Department of Conservative Dentistry, Seoul St. Mary’s Dental Hospital, College of Medicine, The Catholic University of Korea, Banpo-daero 222, Seocho-gu, Seoul, 06591 Republic of Korea

**Keywords:** Characteristics, Cracked teeth, Periodontal probing depth, Pulp status

## Abstract

**Background:**

The purpose of this study was to analyze the characteristics of cracked teeth and to evaluate pulp status according to periodontal probing depth (PPD).

**Methods:**

A total of 182 cracked teeth were included. The location and type of the cracked teeth, age and gender of the patients, restoration type, pulp status, PPD, and radiographic findings were analyzed.

**Results:**

Mandibular second molars (25.3%) were the most frequently involved teeth, followed by mandibular first molars (22.5%), maxillary first molars (22.0%), and maxillary second molars (17.6%). The patient age was most frequently 50–59 years. Cracks occurred mainly in nonbonded restorations, such as gold (26.9%), and were usually found in intact teeth (37.9%). A total of 103 teeth (56.6%) had an initial PPD of less than 3 mm, while 40 (22.0%) had a PPD of 4–6 mm, and 39 (21.4%) had PPD of 7 mm or more. A total of 33 cracked teeth (18.1%) were diagnosed with pulp necrosis, 40 (22.0%) with irreversible pulpitis, and 97 (53.3%) with reversible pulpitis. The incidence of pulp necrosis was 31.8% among cracked teeth with a PPD of 4–6 mm, and 28.6% among those with a PPD of 7 mm or more.

**Conclusions:**

Cracks occurred mainly in molar teeth, and were commonly found in intact teeth with no restoration. Patients with cracked teeth were most frequently aged 50–59 years. Cracked teeth showing a PPD of more than 4 mm were more likely to show pulp necrosis.

## Background

A cracked tooth is defined as an incomplete fracture initiated from the crown, extending subgingivally and usually directed mesiodistally. The fracture is sometimes located in the crown portion of the tooth only, but may also extend from the crown to the proximal root. Occlusally, a crack is more centered and apical than a fractured cusp and therefore more likely to cause pulpal and periapical pathosis as it extends apically [1–4]. According to Ellis [5], a crack can be defined as “a fracture plane of unknown depth and direction passing through the tooth structure that may progress to communicate with the pulp and periodontal ligament.”

Cracked teeth may result in acute pain on mastication and early brief pain with exposure to cold. Cracks are also associated with normal to deep periodontal probing, and no detectable movement of the cusp with an explorer. The teeth may have been restored, where removal of any existing restoration could be required to definitively diagnose the crack [6, 7]. Pain upon loading on the cusp can be explained by dentinal fluid flow caused by movement between fracture sites [8, 9].

A pulpal and periapical diagnosis is dependent on the extent of the crack and duration of the symptoms [10]. The pulp of a cracked tooth might become inflamed because of microleakage, which induces thermal sensitivity. Crack propagation can eventually lead to irreversible pulpitis [11]. Once the crack has extended and exposed the pulp, severe pulp and periapical pathosis will likely occur [12–15]. In addition, extension of the crack can cause a bony dehiscence with a resulting narrow and deep periodontal pocket and/or extensive periapical bone resorption [16, 17]. Treatment options for cracked tooth were direct bonded composite resin, indirect resin or ceramic inlay, full coverage crown, and root canal treatment. A severely cracked tooth may need extraction [11, 18, 19].

The purpose of this study was to analyze the various factors associated with cracked teeth and evaluate the pulp status according to periodontal probing depth (PPD).

## Methods

The protocol for this study was approved by the Institutional Review Board of Seoul St. Mary’s Dental Hospital, The Catholic University of Korea, Seoul, Korea (KC14RISI0240). Patients included in this study 1) were referred from local clinics for treatment of a cracked tooth or visited our clinic with acute symptoms on mastication, 2) had a tooth in which a visible crack line was found, 3) were diagnosed with a cracked tooth via methylene blue dye, a microscope, or a transilluminator, or 4) had a tooth in which a crack line was observed during operative or endodontic treatment. We excluded cracked teeth that were affected by chronic periodontitis. A total of 182 cracked teeth, of patients treated in the Department of Conservative Dentistry, Seoul St. Mary’s Dental Hospital, Seoul, Korea were screened and evaluated from July 2011 to March 2014. Informed consent was obtained from each patient on the day of diagnosis of the cracked tooth.

General and pretreatment data for the cracked teeth were obtained from clinical records. Patient age and gender, type and location of the tooth, location of the crack, PPD, presence and type of restoration, pulp status, and radiographic findings were all noted. Pulp vitality testing of the tooth was done with Endo-Ice (Frigi-Dent™ thermal pulp tester; Ellman International, Inc., Hicksville, NY, USA) and electronic pulp tester (EPT) (Digitest™ tooth vitality tester; Parkell, Inc., Edgewood, NY, USA) because these tests can reveal pulpal pathology. If the cracked tooth showed no response to Endo-Ice, we used EPT to confirm the diagnosis of pulp necrosis. A clinical examination was performed, and periapical radiographs were taken, to confirm the pulp and periapical status. All clinical examinations were evaluated by two faculty members of the Department of Conservative Dentistry. When a cracked tooth was identified, the tooth was anesthetized, and the restoration removed if present. This allowed for thorough visual inspection to identify the position and extent of the crack. Dyes, microscopes, and transillumination served as useful guides. We used methylene blue dye in all cases of cracked teeth. If a definite crack line was not detected using methylene blue, we then used a microscope (Zeiss OPMI Pico; Carl Zeiss, Oberkochen, Germany) and transilluminator (Q-ray view; All in One Bio, Inc., Seoul, Korea). If a crack line was still not detected, the tooth was not included in the study.

Reversible pulpitis was diagnosed if patients showed cold sensitivity lasting a few seconds and biting pain. Irreversible pulpitis was diagnosed if there was prolonged pain on the cold test. Pulp necrosis was diagnosed if there was no response on the cold and EPT tests and/or a periapical lesion was observed on periapical radiographs.

The null hypotheses were as follows: 1) there will be no difference in crack incidence according to patient age, patient gender, tooth type, tooth location, or restoration type; and 2) there will be no difference in pulpal pathology according to PPD. Fisher exact tests and Mantel-Haenszel chi-square test were performed to test the hypotheses and all statistical analyses were done using SAS software (ver. 9.2; SAS Institute Inc., Cary, NC, USA). The level of significance was set at *p* = 0.05.

## Results

A total of 182 cracked teeth were included in this study. The factors associated with cracked teeth are presented in Table 1. Mandibular second molars (25.3%) were the most frequently cracked teeth, followed by mandibular first molars (22.5%), maxillary first molars (22%), and maxillary second molars (17.6%). However, there was no statistically significant difference among posterior molars (*p* > 0.05). The patients were most frequently aged 50–59 years (36.8%). Sixty-nine cracked teeth (37.9%) were intact with no restoration. Among the restored teeth, cracks occurred mainly in nonbonded restorations, such as gold (26.9%), followed by nonbonded restorations such as amalgam (14.3%), and bonded restorations such as resin (11.5%). There was no statistically significant difference between male and female in the crack occurrence (*p* > 0.05).

**Table 1:** Characteristics of the cracked teeth

	Total, *n* (%)	Significance on Fisher’s exact test
Location of tooth		*p* < 0.05
Maxillary second molar	32 (17.6) ^a^	
Maxillary first molar	40 (22) ^a^	
Maxillary second premolar	9 (4.9) ^b, c, d^	
Maxillary first premolar	9 (4.9) ^b, c^	
Mandibular third molar	1 (0.5) ^d, e^	
Mandibular second molar	46 (25.3) ^a^	
Mandibular first molar	41 (22.5) ^a^	
Mandibular second premolar	2 (1.1) ^c, d, e^	
Mandibular first premolar	2 (1.1) ^c, d, e^	
Patient age (years)		*p* < 0.05
20–29	8 (4.4) ^f, g^	
30–39	39 (21.4) ^h, i^	
40**–**49	37 (20.3) ^h, i^	
50–59	67 (36.8) ^j^	
60–69	20 (11.0) ^f, h, i^	
70–79	11 (6.0) ^f, g, i^	
Patient gender		*p* > 0.05
Male	101 (55.5) ^k^	
Female	81 (44.5) ^k^	
Restoration type		*p* < 0.05
Gold inlay	49 (26.9) ^l,^	
Amalgam	26 (14.3) ^m^	
Resin	21 (11.5) ^m, n^	
Temporary filling	17 (9.3) ^n^	
No restoration	69 (37.9) ^o^	

Different superscript letters indicate significant differences at *p* < 0.05

A total of 103 teeth (56.6%) had a pretreatment PPD of less than 3 mm, 40 (22.0%) had a probing depth of 4–6 mm, and 39 (21.4%) had a probing depth of 7 mm or more (Table 2). A total of 33 cracked teeth (18.1%) were diagnosed with pulp necrosis, and 40 (22.0%) were diagnosed with irreversible pulpitis. The 97 cracked teeth (53.3%) that had cold sensitivity and/or biting pain were diagnosed with reversible pulpitis (Table 3).

**Table 2:** Analysis of the periodontal probing depth of cracked teeth

PPD (mm)	*n* (%)	Significance on Fisher’s exact test
≤ 3	103 (56.6)	*p* < 0.05
4–6	40 (22.0)	
≥ 7	39 (21.4)	
Total, *n* (%)	182 (100)	

*PPD* periodontal probing depth

**Table 3:** Analysis of the pulp status of cracked teeth

Pulp status	*n* (%)	Significance on Fisher’s exact test
Reversible pulpitis	97 (53.3)	*p* < 0.05
Irreversible pulpitis	40 (22.0)	
Pulp necrosis	33 (18.1)	
Previously initiated therapy	12 (6.6)	
Total, *n* (%)	182 (100)	

**Fig. 1:**
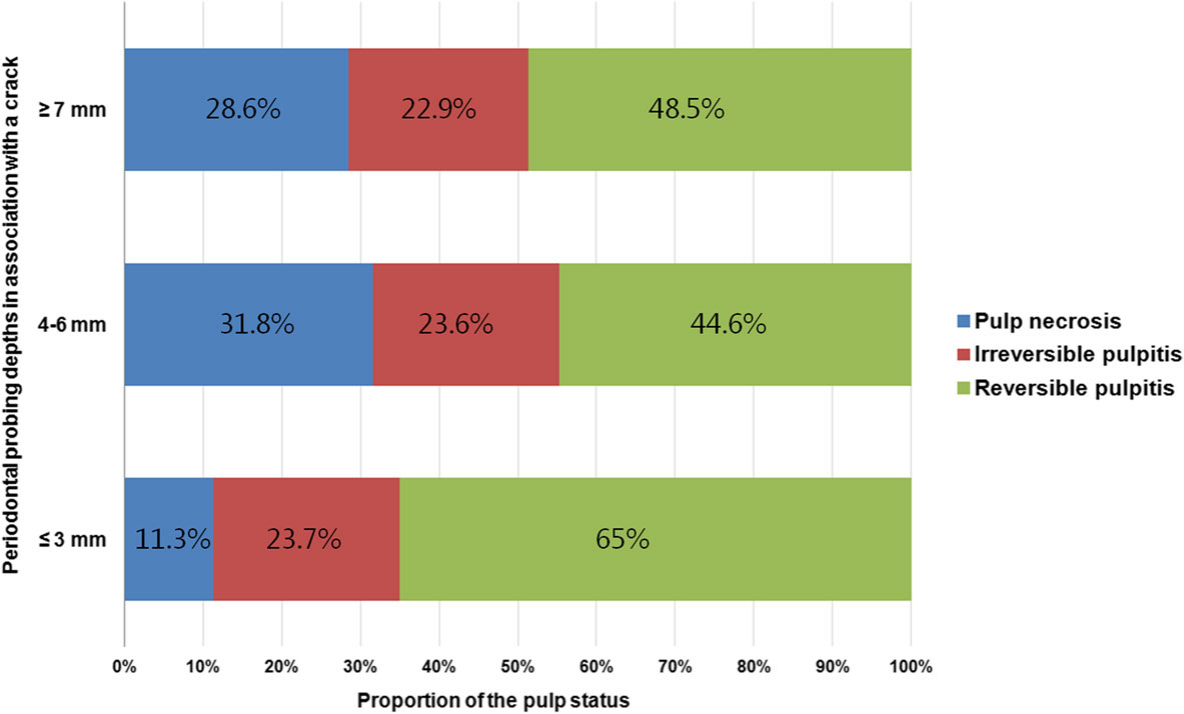
Pulp status of cracked teeth according to periodontal probing depth (*p* for trend <0.05). Pulp status was classified into three categories

The most common pulp status was reversible pulpitis (65%) in cracked teeth with PPD of less than 3 mm, and the proportion of pulp necrosis was low (11.3%). However, if PPD of cracked teeth extended more than 4 mm, the proportion of pulp necrosis increased (31.8% with PPD of 4–6 mm, 28.6% with PPD of 7 mm or more) (*p* for trend <0.05; Fig. 1).

## Discussion

This study analyzed various characteristics and the pulp status of cracked teeth according to PPD. The most frequently cracked teeth were mandibular second molars (25.3%), mandibular first molars (22.5%), maxillary first molars (22%), and maxillary second molars (17.6%). This was similar to previous research in which mandibular molars were the most frequently cracked teeth [2, 3]. The wedging effect of the cusp-fossa relationship has been considered a primary factor in cracked teeth. Many studies [10, 20, 21] have suggested that the palatal cusp of the upper molars acts as a plunger that induces structural fatigue in the lower antagonists. These studies noted that lower molars have deeper central fossa than upper molars, and the oblique ridge of the upper molars provides resistance against crack formation and propagation. However, there was no significant difference in the prevalence of cracks among different posterior molar teeth (*p* > 0.05), and maxillary and mandibular molars showed a higher prevalence of cracks than premolars (*p* < 0.05) (Table 1).

Age is also related with the occurrence of cracked teeth. The resistance of human dentin to fatigue cracks decreases with both age and dehydration. Bajaj et al. [22] found differences in the microscopic features of the fracture surfaces between old and young dentin. The mechanisms contributing to energy dissipation and crack growth resistance in young, hydrated dentin were not present in old dentin. In this study, cracked teeth were most often reported in people aged 50–59 years (Table 1). No difference was found in the number of cracked teeth between males and females, which is similar to other studies using Korean populations [3, 4, 23].

**Fig. 2:**
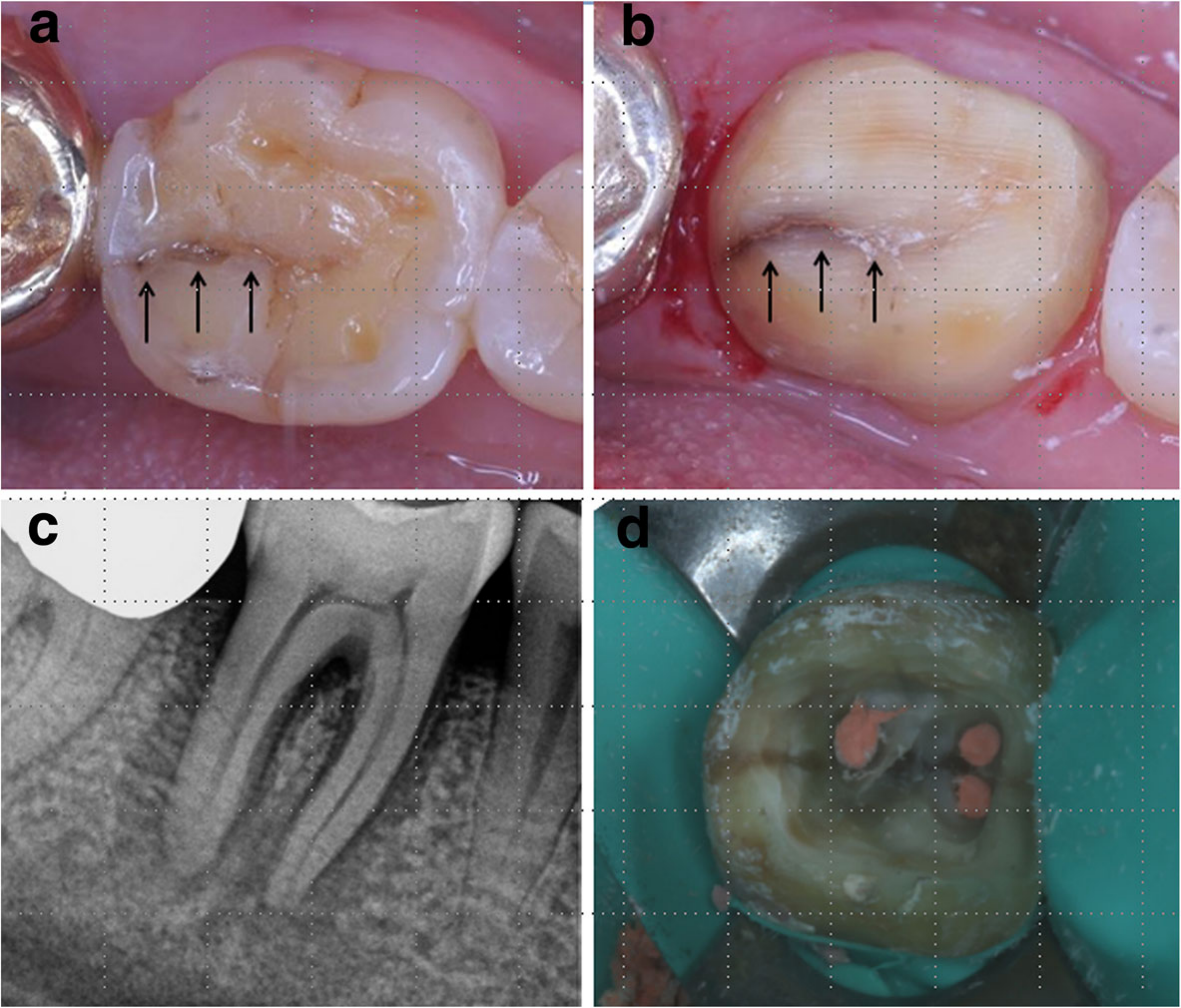
Crack that developed in a mandibular first molar with no restoration. **a**, **b** A distal crack line (black arrows) was observed in the central groove of the mandibular first molar. **c** Periapical lesion was detected in the periapical radiograph. **d** During an endodontic procedure, a crack line extending to the pulp chamber floor was seen

In this study, more than one-third of all cracks occurred in teeth with no restoration (37.9%). Figure 2 shows images of a distal crack that developed in a mandibular first molar with no restoration and a diagnosis of pulp necrosis. There have been several studies reporting a high incidence of cracks among intact teeth [2, 3, 23]. Trauma from occlusion and parafunctional habits might be responsible for the progression of cracks in intact teeth [5]. However, parafunctional habits and occlusion forces were not evaluated in this study; this may constitute a limitation. Among the restored teeth, the greatest number of cracks was seen among gold-restored teeth (26.9%) followed by those restored with amalgam (14.3%) and resin (11.5%) (Table 1). Cracked teeth may readily result from non-bonded heavy restorations and the sharp internal line angles associated with gold restoration. Previous research has suggested that microcracks form in teeth with a non-bonded restoration, as a result of cusp flexure caused by occlusal load stress during mastication and repeated thermal expansion of the restorative materials [4]. In contrast, occlusal stress can be distributed through the bonding layer in a bonded-type restoration, so that the prevalence of cracks is reduced [24]. In this study, several complex factors, such as patient age, tooth type, and presence and type of restoration, were associated with the occurrence of cracked teeth.

A total of 56.6% of the cracked teeth showed a PPD of less than 3 mm; however, 43.4% showed a deep PPD of 4 mm or more (Table 2). All PPDs in this study were isolated and not associated with chronic periodontal disease. They were probably caused by fine cracks that propagated over time, undermining support of the periodontium. Ricucci et al. [25] found that bacteria from the biofilm invade the tissues beneath the crack line depending on the crack’s direction and extent. The response of the pulp to bacterial colonization of the crack varies according to the depth of the bacterial infection. According to Abbott and Leow [26], the symptoms of cracked teeth vary depending on the state of the pulp and periapical disease, and the extent and position of the crack.

The term “cracked tooth syndrome” is misleading as there are a variety of symptoms that together do not constitute a distinct or consistent pattern. There is significant symptomatic variation between teeth with healthy pulp, teeth with inflamed or necrotic pulp, and teeth that have been treated endodontically [27]. Cracks can be seen both symptomatic and asymptomatic teeth and are an etiological factor in pulpal disease. Crack extension causes direct bacterial infestation or microleakage of bacterial toxins indirectly into the pulp chamber [8, 28, 29]. In this study, the proportion of cracked teeth with irreversible pulpitis was 22.0%, while 18.1% showed pulp necrosis. A possible explanation for the high prevalence of irreversible pulpitis and pulp necrosis in cracked teeth among the Korean population is the preference for hot and fibrous/hard food. Brown et al. [30] showed that severe cracking, or the propagation of existing cracks, develops in extracted teeth due to thermal cycling. The clinic where this study was performed is located at a university hospital; therefore, many patients are referred to an endodontist with spontaneous pain or prolonged symptoms. This is also thought to be a possible reason for the high prevalence of irreversible pulpitis and pulp necrosis among cracked teeth.. However, laser Doppler flowmetry (LDF) was not used in this study. LDF has been shown to be reliable for measuring pulpal blood flow. This might be a limitation of this study.

In this study, cracked teeth showing a PPD of 4 mm or more were more likely to show pulp necrosis than cracked teeth with a PPD of less than 3 mm (*p* for trend <0.05; Fig. 1). Periodontal pockets can act as pathways for further infection, resulting in the loss of pulp vitality. A total of 33 cracked teeth were diagnosed with pulp necrosis, among which 13 had periapical lesions (data not shown). No dental caries had advanced into the pulp, and no secondary caries had occurred under the restoration. Therefore, in these cases, cracks rather than caries were most likely responsible for the pulp necrosis and symptomatic apical periodontitis or abscess. In a previous study, cracks were regarded as the main route of pulp infection in traumatized teeth with necrotic pulp and apparently intact crowns [31].

Teeth with cracks that involve both marginal ridges, extend vertically through the pulp, or involve the subpulpal floor have a poor prognosis [12, 15, 32]. A deep PPD suggests that the crack has progressed deep into the root surface. According to Berman and Kuttler [15], the prognosis for teeth having “fracture necrosis,” i.e., pulp necrosis secondary to a longitudinal fracture extending from the occlusal surface into the pulp, may be poor and extraction could be the best treatment option. Since splitting can occur after cracking, early detection of a cracked tooth will facilitate correct treatment and aid prevention of this unwanted consequence [27, 33, 34].

As more patients now retain their teeth into older age, the prevalence of cracked teeth is likely to increase in the future. It is difficult to predict the prognosis for cracked teeth because there is no accurate way to know how advanced the crack has become [35]. This condition has always presented a restorative dilemma for dentists, because a crack has an unpredictable prognosis. Therefore, clinicians must understand the characteristics of cracked teeth and apply appropriate treatment approaches according to the pulp and periodontal status. Furthermore, long-term follow-up is necessary for full evaluation of the prognosis of cracked teeth.

## Conclusions

In this study, cracks occurred mainly in molar teeth, and were commonly found in intact teeth with no restoration. Patients with cracked teeth were most frequently aged 50–59 years. Cracked teeth showing a PPD of 4 mm or more were more likely to show pulp necrosis than those with a PPD of 3 mm or less. Clinicians must be cognizant of the characteristics of cracked teeth and evaluate both the pulp and periodontal status for proper management.

## Abbreviations

EPT: Electronic pulp tester
PPD: Periodontal probing depth
